# Exploration of Factors Influencing Participation of Primary Eye Care Clinicians in Low Vision Services

**DOI:** 10.1007/s44402-026-00037-z

**Published:** 2026-03-13

**Authors:** Gemma Gould, Simon Read, Christine Dickinson, Robert Harper

**Affiliations:** 1 Faculty of Biology, Medicine and Health, University of Manchester, Manchester, UK; 2 NIHR Health Determinants Research Collaboration (HDRC) Torfaen, Torfaen County Borough Council, Wales, UK; 3 Manchester Royal Eye Hospital, Manchester University NHS Foundation Trust, Manchester, UK

**Keywords:** Behaviour change theory, COM-B, Low vision, Primary care optometry, Theoretical domains framework

## Abstract

**Purpose:**

Low vision (LV) services are likely to become more in demand as the prevalence of vision impairment increases. Primary eye care clinicians represent a substantially underutilised resource for LV service provision and increasing their participation could considerably improve service capacity and accessibility. This study aimed to gain a comprehensive in-depth theory-based understanding of factors influencing participation of primary eye care clinicians in LV services to inform the evidence base for future behaviour change intervention design.

**Methods:**

Semi-structured one-to-one interviews using topic guides based on the capability, opportunity, motivation – behaviour (COM-B) system were conducted with a maximum variation sample of primary eye care clinicians and other relevant stakeholders. Thematic data analysis was undertaken; codes were inductively generated then mapped to domains of the theoretical domains framework (TDF) to generate themes and subthemes, which were mapped back to the COM-B system.

**Results:**

A total of 31 individual interviews were conducted. Multiple TDF domain themes and subthemes were found to influence primary eye care clinicians’ participation in LV services, including knowledge (knowledge gaps); memory, attention and decision processes (case identification); social influences (professional support and influences, clinician-patient relationships, interprofessional relationships); environmental context and resources (funding and commissioning, practice resources); intentions (passion); beliefs about consequences (LV outcome expectations); beliefs about capabilities (confidence); goals (profitability); professional role and identity (scope of practice); reinforcement (rewards) and emotion (enjoyment, clinician wellbeing).

**Conclusion:**

This is the first study to qualitatively explore factors influencing participation of primary eye care clinicians in LV services and to explicitly apply behaviour change theory to do so. It provides a novel, comprehensive, in-depth and theory-based understanding of influences on primary eye care clinicians’ participation in LV services. This evidence base is fundamental to designing successful theory-informed behaviour change interventions which aim to increase primary eye care clinicians’ participation and facilitate LV service expansion.

Key Points

- Primary eye care clinicians currently represent a substantially underutilised resource for low vision service provision; increasing their participation could improve service capacity and accessibility considerably.
- Interconnected factors relating to the theoretical domains framework link to an individual’s capability, opportunity and motivation and appear to determine participation in low vision services.
- A comprehensive in-depth understanding of determinants of primary eye care clinicians’ participation in low vision services is required to inform the evidence base for a theory-informed approach to intervention design.

## Introduction

The prevalence of blindness and sight loss in the UK is projected to increase in a policy neutral environment from 3.0% of the population in 2013 to 5.4% of the population by 2050 [1]. Vision impairment (VI) can negatively impact those affected and can lead to activity limitations and participation restrictions [2]. Low vision (LV) rehabilitation services aim to reduce the negative impact of VI and positive outcomes may include increased independence, decreased burden of care, ability to return to a previous role/occupation and improved quality of life [3]. LV services are likely to become more in demand as the prevalence of VI increases.

A large UK survey conducted over 25 years ago indicated that primary care optometry practices made up a very small proportion of LV service provision despite being the largest potential provider group, and the majority of LV services were provided by hospitals [4]. Notably, LV services were unevenly distributed and had insufficient capacity to support everyone who may benefit [4]. In 2004, Wales introduced a national LV service based in primary care practices, but elsewhere in the UK commissioned primary care LV services remain relatively few [5]. A 2017 review of LV service provision in Scotland found that access to LV services was still inequitable, with a scarcity of services in rural areas and variable waiting times [6]. Further, a 2023 UK-wide survey found that the majority of services were still based in hospitals and inequalities in access to support persisted [7].

Primary care optometry practices likely represent a significantly underutilised resource for LV service provision. The positive impact of expanding LV service provision in primary care is demonstrated by the LV service in Wales, whereby its introduction increased capacity by over 50%; reduced waiting times from over 6 months to less than 2 months for most people; reduced journey times to appointments [8], and attained positive patient reported outcomes 18-months post-attendance [9].

Multiple surveys internationally have attempted to identify optometrists perceived obstacles to providing LV services [10–13]. The surveys included a relatively narrow range of stakeholders and span countries which differ in their economic status, service delivery models and training requirements. Moreover, there is a paucity of qualitative data about factors influencing provision of LV services by primary eye care clinicians.

Behaviour change theory is recommended when considering interventions to change a target behaviour [14], and has been relatively widely used in healthcare research [15]. While investigation of eye care clinicians using behaviour change theory is currently limited to a fairly small number of studies [16–20], it is likely to be valuable when considering how to increase primary eye care clinicians’ participation in LV services. The capability, opportunity, motivation-behaviour (COM-B) system is used to understand mechanisms of behaviour change whereby an individual’s capability (psychological and physical), opportunity (social and physical) and motivation (reflective and automatic) interact to influence behaviour, which in turn influences those components [21]. The theoretical domains framework (TDF) can be mapped onto the COM-B system and aims to simplify and consolidate multiple behaviour change theories to provide a comprehensive accessible approach to assess influences on healthcare workers’ behaviour [14, 22]. Understanding influences on behaviour provides an evidence base for a theory-informed approach to behaviour change intervention design [21].

This study aimed to gain a comprehensive in-depth, theory-based understanding of factors influencing participation of primary eye care clinicians in LV services in the UK. Such an understanding is essential to facilitate theory-informed design of effective interventions to expand LV service provision in primary care, and to manage the pressing need for improvements to LV service capacity and accessibility.

## Methods

The study was designed and reported according to the Consolidated Criteria for Reporting Qualitative Research [23].

### Study Design and Ethical Approval

This study used qualitative methodology with semi-structured one-to-one interviews. A letter was issued from the University of Manchester Research Ethics Team on 23 February 2024 to confirm the study was granted exemption from formal ethical review under the category of work with professionals (Ref: 2024-19478-33316). The study was conducted in accordance with the standards of the Declaration of Helsinki.

### Recruitment

Purposive sampling was used to recruit participants with diversity in location, years of work experience, job role and setting, experience in LV service provision and attainment of postgraduate LV qualifications. Inclusion criteria were optometrists and dispensing opticians (DOs) registered with the UK General Optical Council and other stakeholders involved in either delivering and/or planning or organising primary eye care services and/or care of patients who have a VI. Participants had to be aged 18 years or older, able to communicate in English or provide a suitable interpreter and able and willing to participate in an online interview. Participants were identified from a previous LV survey [24] and via the professional networks of the research team. All participants were invited to participate and sent a participant information sheet via email. Nobody explicitly refused to participate or dropped out, but several individuals did not respond to the invitation email.

### Data Collection

Participants had the opportunity to ask questions before signing an electronic consent form on the Qualtrics XM platform (Qualtrics.com). Data were collected between May 2024 and August 2025. Interviews were conducted via Microsoft Teams (Microsoft.com) by a female PhD researcher and optometrist experienced in LV rehabilitation and trained in qualitative research (GG). Interviews were audio and video recorded and transcribed on Microsoft Teams.

Semi-structured one-to-one interviews used topic guides based on the COM-B system (included in Supplement A). The topic guide was pilot tested with two optometrists who work in primary care and then refined prior to data collection. Interviews opened with questions about participant characteristics, e.g. UK region, years since qualification (if applicable), postgraduate professional qualifications (if applicable), description of job role(s) and experience with LV and/or planning or organising primary eye care services and/or care of patients who have a VI. Participants were asked about capabilities, opportunities and motivations to participate in LV service provision and specific probing questions were used to prompt discussion about unanticipated areas raised. Field notes were made during interviews and no repeat interviews were undertaken.

The sample size was not predetermined. Participants were recruited and interviews conducted and analysed iteratively until a maximum variation sample had been interviewed and no new themes or explanations emerged from successive interviews, achieving our agreed definition of data saturation [25].

### Data Analysis

Thematic data analysis [26] was undertaken using the qualitative software package NVIVO 12 (lumivero.com/products/nvivo). Data familiarisation was undertaken by GG who checked and edited transcripts against original recordings to ensure accuracy and read and re-read the data. Transcripts were not returned to the participants. GG reviewed data line-by-line and used a grounded theory approach to inductively generate initial codes. Codes were discussed and refined at regular intervals with optometrists CMD and RAH and experienced qualitative researcher SR. After coding 10 interviews there was noticeable alignment between the inductively generated codes and TDF domains, and codes were deductively mapped to the TDF domains to generate themes through multiple discussions with the whole research team. Coding and mapping were refined through further discussions between the whole team, as were reviewing and finalising themes and subthemes. Final TDF domain themes were mapped back to the COM-B system. Participants were not invited to provide feedback on the findings.

## Results

### Participant Characteristics

A total of 31 interviews were conducted. The mean interview duration was 56 min (range 34–127 min). Table 1. summarises the diversity of participant characteristics. Optometric societies/committees included a range of regional and national organisations which represent optometrists/DOs and/or provide professional education.

**Table 1:** Summary of key participant characteristics.

Participant ID	UK region	Years since qualification	Current job role(s) and position(s) held	Type of practice	Participation in LV services	Postgraduate LV qualification^a^
P01	North West	16–20	Optometrist franchise partner	Multiple	Previous	-
P02	South West	6–10	Optometrist	Independent	Nil	-
P03	Northern Ireland	41–45	Optometrist practice owner	Independent	Previous	+
P04	Northern Ireland	26–30	Senior optometrist, optometric society/committee member	Multiple	Nil	-
P05	Wales	6–10	Optometrist	Multiple	Current	+
P06	North East	36–40	Optometrist, optometric society/committee member	Independent	Current	-
P07	Scotland	31–35	Optometrist practice owner	Independent	Current	+
P08	London	21–25	Manager of sight loss charity, optometrist	Charity	Current	-
P09	East Midlands	36–40	Optometrist practice partner	Independent	Nil	-
P10	North East	6–10	Resident optometrist, professional qualification examiner, university clinic tutor	Multiple	Nil	+
P11	West Midlands	0–5	Dispensing optician retail manager	Multiple	Nil	-
P12	North West	n/a	Eye Clinic Liaison Officer	n/a	n/a	n/a
P13	West Midlands	26–30	Dispensing optician practice partner	Independent	Current	+
P14	North West	n/a	Consultant Ophthalmologist Medical Director	n/a	n/a	n/a
P15	South West	11–15	Dispensing optician, optometric society/committee member	Domiciliary	Current	+
P16	North East	26–30	Senior leader of commissioning organisations, optometrist	n/a	Previous	-
P17	North West	n/a	Consultant Ophthalmologist	n/a	n/a	n/a
P18	North East	21–25	Optometrist, optometric society/committee member	Charity	Current	+
P19	North West	n/a	Council Sensory Team manager	n/a	n/a	n/a
P20	Wales	0–5	Optometrist	Independent	Nil	-
P21	London	16–20	University educator, optometrist	University	Current	+
P22	West Midlands	11–15	Locum dispensing optician, professional qualification examiner, optometric society/committee member	Multiple and Independent	Nil	-
P23	North West	6–10	Locum optometrist, university clinic tutor	Multiple	Nil	-
P24	North West	6–10	Dispensing optician	Multiple	Nil	-
P25	Yorkshire and Humber	11–15	Dispensing optician, professional qualification examiner, optometric society/committee member	Independent	Previous	-
P26	North West	11–15	Senior leader of NHS eyecare provider company, optometric society/committee member, locum optometrist	Multiple	n/a	-
P27	North West	11–15	Optometrist director, optometric society/committee member	Independent	Yes	-
P28	Yorkshire and Humber	6–10	Dispensing optician	Independent	Nil	-
P29	East Midlands	21–25	Dispensing optician	Multiple	Previous	-
P30	North West	n/a	Commissioner	n/a	n/a	n/a
P31	North West	0–5	Optometrist	Independent	Nil	-

*LV* low vision, *n/a* not applicable.

^a^+ indicates the participant has achieved a postgraduate LV qualification, - indicates the participant has not achieved a postgraduate LV qualification.

### Themes

**Fig. 1:**
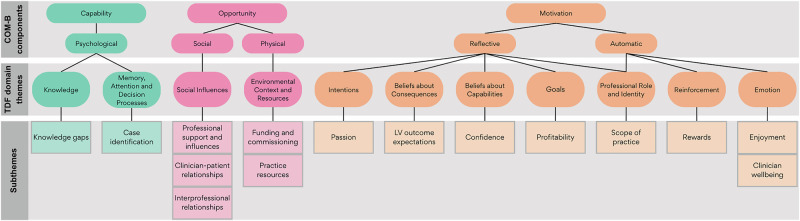
Summary of themes and subthemes. The top panel contains components of the COM-B system, the middle panel contains the TDF domain themes, and the bottom panel contains subthemes. The lines link the COM-B system with the TDF domain themes and subthemes. Summary of abbreviations: COM-B capability, opportunity, motivation-behaviour, TDF Theoretical Domains Framework.

Figure 1 summarises the TDF domain themes and the subthemes and how these link to components of the COM-B system. Illustrative quotes to support the analysis are included in Supplement B and are referenced in the text by numbers in round brackets (i.e. 1.1–15.6).

## Capability

### Knowledge: Knowledge Gaps

A few participants were confident that all eye care clinicians’ LV knowledge should be sufficient to provide LV services because LV was part of their professional education (1.1), yet both optometrists and DOs recognised weaknesses in their own and their peers’ LV knowledge (1.2, 1.3). Specific areas with reported lack of knowledge included awareness of up-to-date product availability and developments in technology (1.4) and awareness of local LV support organisations, which may be challenging because of variability in services between areas (1.5). Optometrists may also have poor knowledge about prescribing high addition spectacles (1.6) and not all DOs know how to measure vision/visual acuity for patients with a VI (1.7). Unrecognised knowledge gaps may include understanding what LV services typically involve, with a DO discussing interpreting Optical Coherence Tomography scans and fundus photos when asked which elements of LV they may lack confidence or knowledge in (1.8).

### Memory, Attention and Decision Processes: Case Identification

Primary eye care clinicians may fail to identify patients who could benefit from LV support, even if they are highly qualified and experienced clinicians (2.1). Clinicians may fail to identify patients because they may focus more on clinical aspects of care rather than holistic support (2.2); LV care may simply not be considered (2.3), and clinicians may assume LV support had already been offered or provided elsewhere if indicated (2.4). Patients appearing less likely to be identified as needing LV support are those with relatively mild VI (2.5) and older age groups who may be less able to self-advocate for support (2.6), despite an appreciation that LV is associated with the elderly (2.7).

## Opportunity

### Social Influences: Professional Support and Influences

Enthusiastic LV educators can influence clinicians’ attitudes towards LV positively by inspiring them with their passion (3.1) and motivating them to help patients achieve their goals (3.2). Having good peer support networks and forums to ask questions may also be strong enablers to providing LV services by offering a sense of community (3.3), as well as professional support (3.4, 3.5). The networks and forums discussed were often informal, such as WhatsApp groups (whatsapp.com), and were welcomed by those who felt they had expertise to share (3.6).

### Social Influences: Clinician-Patient Relationships

Clinicians may feel uncomfortable directing their longstanding patients elsewhere for LV care (4.1), and they may believe that offering LV services could strengthen patients’ loyalties to their practice (4.2). However, if clinicians perceive their patients to be unmotivated to receive LV support, they may feel demotivated to offer it (4.3). Patients may be hesitant to access LV support in primary care owing to worry about pressure to purchase spectacles (4.4), and they may underappreciate primary eye care clinicians’ expertise (4.5).

### Social Influences: Interprofessional Relationships

Multiple participants highlighted the benefits of a multidisciplinary approach to LV care. Eye Clinic Liaison Officers (ECLOs) were frequently cited as having a key role in helping patients with a VI access support (5.1), but notably an ECLO had low confidence that primary eye care clinicians were capable of providing LV services (5.2) and they thought primary eye care clinicians lacked awareness about ECLO roles (5.3). Similarly, a consultant ophthalmologist lacked confidence in primary eye care clinicians’ capabilities to support patients with complex pathologies (5.4). There may also be a longstanding lack of trust from secondary to primary care (5.5), as well as improvements needed in primary eye care clinicians being appropriately recognised for their role in patient care (5.6). Additionally, clinicians discussed a lack of engagement from local sight loss support charities and community groups with optometry practices (5.7), and they questioned the integrity of commercial sector organisations (5.8).

### Environmental Context and Resources: Funding and Commissioning

Securing appropriate funding and commissioning is widely perceived as pivotal for increasing LV service provision in primary care (6.1). That said, a practice with funding and commissioning for LV services has been unable to attract clinicians to participate (6.2). Perceived barriers to securing funding and commissioning for LV services in primary care include there being a general shortage of available funds (6.3); poor awareness among Commissioners and other stakeholders about the need to expand LV service provision (6.4); weak evidence supporting the positive impact of LV services (6.5); LV not being a priority for commissioning review (6.6) and optometry not being a commissioning priority among other healthcare sectors (6.7).

### Environmental Context and Resources: Practice Resources

Access to LV aids and appropriate equipment to assess visual function (7.1, 7.2), sufficient space for equipment storage (7.3) and adequate allocation of time to spend with patients (7.4) were all discussed as both barriers and enablers to LV service provision in primary care. Additionally, poor physical accessibility within practices may be a barrier to patients with a VI accessing care (7.5).

## Motivation

### Intentions: Passion

Passion for LV can drive primary eye care clinicians to participate in LV service provision (8.1) and to advocate for and help to establish local LV services (8.2). Several individuals attributed their passion for LV to personal experiences with family members with a VI or other disability (8.3). However, clinicians who are passionate and seek active involvement in LV services may be in the minority (8.4), and many clinicians either may have no intention to participate (8.5) or a general indifference or lack of interest in LV (8.6).

### Beliefs About Consequences: LV Outcome Expectations

Clinicians discussed how issuing aids and giving simple advice can make big differences to patients (9.1, 9.2); how LV care can help reduce patients’ additional care needs and keep them safe (9.3), and that LV work can feel more impactful than other areas of optometric practice (9.4). Contrastingly, others lacked belief that LV work will have positive outcomes because they feel they will never meet patients’ expectations of restoring vision (9.5); they have low confidence that aids will benefit their patients (9.6) and they believe patients may not be willing to accept their advice (9.7).

### Beliefs About Capabilities: Confidence

Low confidence in LV may be a significant barrier to participation in LV service provision (10.1). Whilst multiple participants expressed a general perception that LV is challenging (10.2), not all see it as a difficult area (10.3). Clinicians’ confidence may be shaped by their experiences at university where LV may have been perceived as a difficult subject (10.4) and may have felt overwhelming because it is different to other clinical areas (10.5). Some clinicians attributed high confidence to previous exposure to a wide range of patients (10.6), while others attributed low confidence to infrequent exposure to LV patients (10.7). Undertaking a postgraduate qualification in LV may improve clinicians’ confidence, although not necessarily to the extent that they feel confident enough to provide LV services in practice (10.8).

### Goals: Profitability

The profitability of providing LV services is perceived as an important factor in terms of how it aligns with the overall business goals of optometric practices (11.1). The poor profitability of LV services compared with other areas may be a key reason for them not being prioritised (11.2). An optometrist participating in the Welsh LV service discussed that their practice was phasing out the scheme solely due to poor profitability (11.3). However, a practice director and LV service provider recognised that although LV services are not directly profitable, they help build a practice’s patient base and align with other goals of supporting patients with a VI (11.4). Moreover, from an ethical standpoint a few clinicians expressed a strong belief that it is inappropriate to benefit financially from patients with a VI (11.5).

### Professional Role and Identity: Scope of Practice

LV is perceived by several primary eye care clinicians as a fundamental part of their role, even if they do not currently participate in LV service provision (12.1, 12.2). Contrastingly, others simply view LV as something they signpost to and make onwards referrals for (12.3), and they may believe LV services should be based in hospitals (12.4). However, a middle ground was often discussed whereby some, but not all, elements of LV were perceived to fit well within primary care. Such elements include a straightforward case mix of patients (12.5) and tasks such as refraction, prescribing high addition spectacles, advising about lighting and technology, and signposting to other support organisations (12.6). Dispensing simple magnifiers was often but not always included as a role fitting into primary care practice and an optometrist expressed that magnifiers fit less well than spectacle aids in primary care settings (12.7). Overall, there was consensus that hospitals should still have a role in providing some level of LV care even if primary care LV service provision increased, mostly for patients with more complex needs (12.8).

### Reinforcement: Rewards

LV can be intrinsically rewarding because clinicians may feel good helping patients who have been struggling (13.1, 13.2) and the sense of reward they get from undertaking LV may be stronger than other areas of practice (13.3). LV can also feel rewarding because it provides clinicians with opportunities to make fuller use of their skills to have a positive impact (13.4). Additionally, clinicians feel rewarded when they are appreciated by patients and their relatives (13.5).

### Emotion: Enjoyment

Clinicians expressed a strong sense of enjoyment from providing LV services (14.1). They enjoy the problem-solving element of LV (14.2), as well as interacting with patients on a more personal level (14.3) and having the opportunity to really help people (14.4). Contrastingly, LV may be viewed as less exciting than other areas of optometric practice (14.5) and some clinicians do not enjoy LV at all (14.6).

### Emotion: Clinician Wellbeing

Clinicians described feelings of anxiety and worry that they may not have provided enough support for their patients (15.1) or that they might make mistakes (15.2). Hearing about patients’ challenging circumstances may negatively impact clinicians’ wellbeing, especially in the early stages of their careers (15.3). Clinicians may learn to deal with this emotional impact better as they become more experienced (15.4) and strategies such as spreading out their LV workload to reduce the emotional burden and discussing issues with peers may help (15.5, 15.6).

## Discussion

To our knowledge, this is the first study to qualitatively explore factors influencing participation of primary eye care clinicians in LV services and to explicitly apply behaviour change theory to do so. It is also the first UK study to explore factors influencing participation in LV service provision. Multiple TDF domains were found to influence primary eye care clinicians’ participation in LV service provision and can be linked to components of the COM-B system.

Several factors influencing participation in LV service provision identified in this study echo the barriers reported from previous surveys in other countries [10–13, 27]. However, this study identified a broader range of relevant factors, likely because a wider range of stakeholders were included and qualitative semi-structured interviews were used rather than surveys with mostly pre-determined options. In keeping with these findings, a systematic review of determinants to optometrists’ delivery of eye care similarly noted a greater diversity of barriers and facilitators reported in qualitative compared with quantitative studies [19]. Additionally, use of the COM-B system to design the topic guide was likely significant in facilitating identification of a broad range of determinants, since it includes and aims to equally emphasise both intrinsic and external factors [21].

The present study also provides greater depth of understanding than previous work. For example, a previous survey of UK optometrists identified that optometrists who do not work in a LV service have relatively low confidence in LV [24] and this study describes underlying factors influencing confidence, such as clinicians’ experiences at university and their previous exposure to LV patients. Additionally, previous studies offered relatively vague reasons for non-participation in LV services, like lack of motivation [11, 13], while this study explores multiple influences on clinicians’ motivation by using the TDF.

The diversity of participants interviewed is a key strength of this study and is highlighted by the broad range of insights obtained, but the possible underrepresentation of devolved UK nations should be acknowledged. That said, some of the insights gained may be universally applicable across different settings, particularly those relating to motivation, and it was beyond the scope of this study to explore potential differences between the devolved UK nations.

The COM-B system is at the centre of the behaviour change wheel framework for characterising and designing interventions to change a target behaviour [21]. We recommend the evidence base provided in this study be used alongside the behaviour change wheel to systematically identify intervention functions and supporting policies to facilitate increased primary eye care clinicians’ participation in LV services. Relevant intervention functions include education, persuasion, incentivisation, coercion, training, restriction, environmental restructuring, modelling and enablement [21].

Successful interventions may need to be multi-faceted and simultaneously address several COM-B components to have maximum impact. The potential for limited impact if only a single component is targeted is highlighted by the participant whose practice has a commissioned LV service yet struggles to attract clinicians to participate in it, and  the clinician who perceives LV to be a fundamental part of their professional role and yet does not provide LV services. That said, factors influencing participation are clearly interconnected and interventions targeting one component are also likely to impact other components. For example, having good peer support networks and forums to ask questions (opportunity) is likely to positively impact clinicians’ LV knowledge (capability) and confidence (motivation). Such interactions may lessen or amplify the impact of interventions so should be carefully considered [21].

Clinicians recognised weaknesses in identifying patients who may benefit from LV support by reflecting on their own practice and making observations about colleagues. Significantly, as well as impacting clinicians’ abilities to provide LV services, failure to identify patients who may benefit from LV support is also likely to contribute to delays in patients accessing existing LV services. In a study in the Netherlands, patients’ self-advocacy was highlighted as an important facilitator for referral to LV services [28], suggesting clinicians may benefit from prompts to recognise patients’ support needs. Further work should aim to investigate the scale of impact of poor identification of patients who may benefit from LV support, and a timely intervention to address this specific issue may be warranted.

The present study provides a novel, comprehensive and in-depth theory-based understanding of influences on primary eye care clinicians’ participation in LV service provision. As the prevalence of VI increases, the need to improve LV service capacity and accessibility will almost certainly become a more pressing challenge. This study is arguably fundamental in facilitating the design of successful behaviour change interventions to address this important requirement.

## Supplementary Information

Supplement A Topic Guide

Supplement B Illustrative quotes

## Data Availability

The full data set cannot be shared openly to protect participant anonymity.
