# Supplementary material for: Exploration of Factors Influencing Participation of Primary Eye Care Clinicians in Low Vision Services
Source: Ophthalmic Physiol Opt. 2026 Mar 13;46(2):261–7. doi: 10.1007/s44402-026-00037-z (PMC13369622; doi:10.1007/s44402-026-00037-z)
Supplement: Supplementary file 1 — Supplement A Topic Guide [file 44402_2026_37_MOESM1_ESM.docx]

**Supplement A: Topic guide for semi-structured one-to-one interviews**

The topic guide below outlines the proposed questions that will be asked. One-to-one interviews will be expected to take up to 45 minutes. Participants will be encouraged to explain or explore their responses in more detail. Deviations may be made from the guide to develop the questioning if the conversation offers information that may benefit the research study.

At the beginning of the interview the interviewer will:

- Explain the purpose of the project.
- Explain the format of the interview: length, topics to be covered, flexibility in structure.
- Highlight the importance of participants’ responses: their opinions and perspectives are important.
- Reiterate consent procedures and offer opportunity to ask any further questions.

**Guide for questions**

1. **Participant characteristics:**
   - UK region
   - Length of time since qualification (if applicable)
   - Postgraduate professional qualifications (if applicable)
   - Current job role
   - Type of optometry practice (if applicable)
   - Current involvement or non-involvement in low vision services / planning or organising primary eye care services and/or care of patients who have a vision impairment
   - Relevant previous job role(s)
2. **Description of previous and current role(s) and experience with low vision / planning or organising primary eye care services and/or care of patients who have a vision impairment:**

Clinicians who work in a low vision service:

- - - What areas of clinical practice do you work in?
    - Describe your previous and current involvement in low vision services.
    - Describe any previous learning relevant to low vision.

Clinicians who do not work in a low vision service:

- - - What areas of clinical practice do you work in?
    - Describe any previous learning or experience relevant to low vision.
    - If you see patients with a vision impairment what, if any, support do you offer?

Wider stakeholders:

- - - Describe your current role in planning or organising primary eye care services and/or care of patients who have a vision impairment.
    - Describe your background or previous experience with planning or organising primary eye care services and/or care of patients who have a vision impairment.

1. **Capability:**

Clinicians who work in a low vision service:

- - - To what extent do you feel confident you have the knowledge and skills for working in a low vision service?
    - What has impacted your professional confidence in low vision?
    - Do you have any plans or intention to further your knowledge and skills relevant to low vision?

Clinicians who do not work in a low vision service:

- - - To what extent do you feel confident you have the knowledge and skills for working in a low vision service?
    - What has impacted your professional confidence in low vision?
    - Do you have any plans or intention to further your knowledge and skills relevant to low vision?

Wider stakeholders:

- - - To what extent do you think primary eye care clinicians have the knowledge and skills to work in a low vision service?
    - Do you think qualified eye care clinicians should have to demonstrate minimum capability requirements to offer low vision services?

1. **Opportunity:**

Clinicians who work in a low vision service:

- - - What are the main influences on allocation of resources in your practice?
    - How does your practice provide the necessary resources (e.g. time, space, equipment) to enable you to provide low vision services?
    - Do you know of other eye care clinicians offering low vision services in primary care? Does this influence your thoughts about providing low vision in primary care?

Clinicians who do not work in a low vision service:

- - - What are the main influences on allocation of resources in your practice?
    - To what extent could your practice provide the necessary resources (e.g. time, space, equipment) to enable you to provide low vision services?
    - Do you know of other eye care clinicians offering low vision services in primary care? Does this influence your thoughts about providing low vision in primary care?

Wider stakeholders:

- - - What are main influences on allocation of resources in optometry practices?
    - To what extent do optometry practices have the necessary resources (e.g. time, space, equipment) to enable them to provide low vision services?
    - Within your professional role, how and to what extent could you influence the provision of primary care optometry services?

1. **Motivation:**

Clinicians who work in a low vision service:

- - - What led you to get involved in low vision work?
    - How does your low vision work make you feel?
    - Would you like to continue working in low vision in the future?
    - How important is your work in low vision compared to other areas of clinical practice?

Clinicians who do not work in a low vision service:

- - - If the opportunity was available, would you want to work in a low vision service?
    - How has previous learning or experience relevant to low vision / seeing patients with a vision impairment made you feel?
    - To what extent do you think low vision falls within the role of primary eye care clinicians?
    - If you specialise in other area(s) of clinical practice, what led you to specialise in those areas?

Wider stakeholders:

- - - How important do you perceive low vision service expansion as a priority for eye care?
    - To what extent could increasing provision of low vision services in primary care benefit patients / eye care clinicians?
    - Are there other solutions which could more effectively improve low vision service availability and accessibility?
