# Supplementary material for: Exploration of Factors Influencing Participation of Primary Eye Care Clinicians in Low Vision Services
Source: Ophthalmic Physiol Opt. 2026 Mar 13;46(2):261–7. doi: 10.1007/s44402-026-00037-z (PMC13369622; doi:10.1007/s44402-026-00037-z)
Supplement: Supplementary file 2 — Supplement B Illustrative quotes [file 44402_2026_37_MOESM2_ESM.docx]

**Supplement B: Illustrative quotes to support the data**

| **Number** | | **Participant** | **Quote** |
| --- | --- | --- | --- |
| **Knowledge: Knowledge gaps** | | | |
| 1.1 | | P05 | I think we've got the basis for it. Everyone's got the knowledge to do it. We all did at university…. So there's no excuse that anyone couldn't do it from a knowledge point of view. |
| 1.2 | | P24 | This customer now has gone out unhappy and probably thinks the lady in XXX hasn't helped me but really it's not about that, it's just because I don't know much about it. |
| 1.3 | | P23 | And then there is maybe I think lack of knowledge. It's like some people maybe they don't understand it fully, maybe they don't know what they can do for this patient. Maybe there are limitations in what they actually know rather than pathways and all that kind of stuff. |
| 1.4 | | P02 | what is the state of magnifiers and CCTVs and things like that at the moment, like what is available to us? Because what's certainly one of the barriers for me in that sense is I might start having that conversation, but I just simply don't really know what's available or kind of even where to get it really. |
| 1.5 | | P16 | I can imagine it would be very challenging for somebody coming into the area to try and get themselves up to speed with that … it's fragmented and it could really do with being coordinated in a way that makes it easier for everybody to understand what's available. |
| 1.6 | | P07 | So when somebody comes in with low vision, and it drives me mad when they're 6/36 and they give them a +2.00 add. You’re going that's a waste of time. So people don't get simple stuff. |
| 1.7 | | P28 | I feel like if it got to the point where they couldn't see 6/60 or 6/120 and I was like ah right, so it's on to hand movements I don’t know… it’s not something that I've ever done before so I don't know how to measure somebody's hand movements. I don't know if it's just light perception or if they've got no vision at all. |
| 1.8 | | P28 | it would be figuring out from like an OCT [Optical Coherence Tomography] scan at what point… especially with glaucoma on an OCT scan on the image with discs, it’s a little bit easier when you've got the thickness chart to see where the thinning is, but if it's just from a fundus photo I don't think that I would be able to pick up on that. |
| **Memory, Attention and Decision Processes: Case identification** | | | |
| 2.1 | | P15 | Oh, it's such a thing. Oh, it's a real thing. I mean, I've come across an optom that’s got glaucoma, IP [independent prescribing], med ret [medical retina], runs a dry eye clinic - couldn't spot a low vision patient. |
| 2.2 | | P10 | But when people see pathology they just focus just on that, rather than the individual and what they actually want to be able to do |
| 2.3 | | P11 | Yeah, that's so bad. I’ve not even thought about it, really. You just get so carried away in what you're actually doing in the high street it does get forgotten, doesn't it? |
| 2.4 | | P01 | So I think everyone just presumes, well, you know you've got macular degeneration, you must have been told this already. So therefore I'll leave it out. Or OK, you've been told you've got a stroke in the hospital and you've been told you've lost the vision because you're not allowed to drive. You must have been told how to cope with it so let me just do a prescription check. There's a lot of presumptions there rather than going back over things to make sure they do know how to work things. |
| 2.5 | | P08 | And the key part of that is who are the patients that are having problems, like how to identify the problems and who might be having them because it's not just the ones that can't see at all or are registered. It's the person that's recently had to give up their driving licence and they can read fine, they can watch TV, but they're not legal to drive for their field or their central vision or whatever it might be, but they need support …. So it's how to identify those people because I think they slip through the net a lot. |
| 2.6 | | P12 | I think an older person is more likely just to keep going to an optician and even if they're told glasses aren't helping you now they wouldn't know to ask questions to say, well, is there anything out there to help me? |
| 2.7 | | P02 | you think about low vision and you do sort of automatically think about the elderly population |
| **Social Influences: Professional support and influences** | | | |
| 3.1 | | P20 | So it was a woman called [name] I mainly had who was really good, really passionate about it which was really helpful…. I think in anything it makes that difference, but especially with low vision because as I said that personal engagement that you need to figure out what's going to benefit each person, if you're not passionate about it then it's not going to be that personal conversation really. |
| 3.2 | | P21 | I think actually, years back when I was a student we used to have a very enthusiastic head of department and he liked low vision. And so people used to refer all these low vision patients to him…. And I think it was because he would always say like just make them try harder, tell them they can do this, and then we sort of got a bit motivated as well. |
| 3.3 | | P15 | I think what I've realised is the people who do low vision really get it and it's almost like a bit of a kindred community. Like as soon as you say someone does low vision then like I've noticed, like each other's eyes light up and go yeah, I get you kind of thing. |
| 3.4 | | P05 | I think it's quite tightly knit as well, so there's big group chats as well where you can ask questions and they've always been quite good. They always pick my calls or call me back…. I think if I didn't have people to ask I wouldn’t have known what to do with the records or where to send them. So yeah, it was really important. |
| 3.5 | | P02 | I think just having that kind of group of people that you could bounce ideas off of or just express concerns with and things like that would be really, really useful in that sort of situation. |
| 3.6 | | P13 | And also we're a pretty small area as such, it's like well if you’ve got a query pick up the phone, send me an e-mail, that's fine. And that's that mature relationship amongst each other. |
| **Social Influences: Clinician-patient relationships** | | | |
| 4.1 | | P05 | I didn't really like sending patients away, especially a few of them I've been seeing for 6-7 years at this stage, and then for me to turn around and say, oh look, you can go elsewhere for this specialist service, it always felt a bit off. So that that was the motivation to do it initially. |
| 4.2 | | P20 | Again, that's just going back to maybe even patients prefer that follow through of not going from one person for the sight test and then a whole other practice for this. If they see that benefit then I think we've seen a few people that have been like now I'll stick to come to the same person who knows about me, knows about how they can help me and things like that. |
| 4.3 | | P24 | it's kind of like they've given up already. I think that's what it is. I think when you get that sort of vibe from them you probably think oh well OK, well if you're not as bothered then why should I be bothered? |
| 4.4 | | P14 | And I think there's definitely a perception that whenever you go to the optometrist, that perhaps there may be some pressure to buy glasses |
| 4.5 | | P09 | Then he said “But you're a glasses seller.” And he didn't just say it once, he just kept going on and on and on. He couldn't believe that I had been to university or that I had had any training whatsoever, because as far as he was concerned I was a shop girl who knew how to flog a pair of specs but knew absolutely nothing about anything else. |
| **Social Influences: Interprofessional relationships** | | | |
| 5.1 | | P16 | it's the ECLO [Eye Clinic Liaison Officer], is that person that coordinates their care. So getting them in touch with an ECLO and to make sure they get those support needs is essential |
| 5.2 | | P12 | no disrespect to opticians in the community, but generally they're not skilled in being able to provide the low vision service…. I think just because they're very focused, understandably, on doing the refractions but not very focused on environmental factors that could impact, they’re not very au fait with how to use the magnifier correctly. Yeah, for me, I just think no. |
| 5.3 | | P12 | I think I have a responsibility as an ECLO [Eye Clinic Liaison Officer] to maybe go and try and communicate more with opticians in fairness because how do they know I'm here, because they don't. |
| 5.4 | | P17 | I don't know how good the optoms there would be to refract and to provide LVAs for really complex pathologies, let's say corneal decompensation and patient has a vitreous haemorrhage. So if they don't understand the clinical aspect are they good enough to manage that situation |
| 5.5 | | P25 | there has historically always been this lack of trust from secondary care to primary care, understandably in some cases, and it will have been so historic that people can't remember why it even started. |
| 5.6 | | P14 | So I think that there's a little bit of work to do as well in terms of just breaking down those barriers and changing convention to make the optometrists a regular co-partner in the care of these patients. |
| 5.7 | | P28 | You'd think that if there was somewhere within the area that they would think oh we’ll drop off a leaflet at the opticians, or we’ll make the opticians aware of a meeting that we might have, but it doesn't seem to happen. |
| 5.8 | | P01 | And that's again another difficult one because when you recommend a company to someone then they go around and they hassle them for a sale it's hard. |
| **Environmental Context and Resources: Funding and commissioning** | | | |
| 6.1 | | P04 | I think if it's not happening it's just because of no NHS funding for it to be in the high street. If there was funding and it was in the contract it would change overnight. |
| 6.2 | | P13 | Some practitioners have stepped out from the practice and those gaps haven't just been filled. … We haven't been able to generate the interest even though we seem to have this fantastic offering. So you can have a great offering but not everyone wants to do it. |
| 6.3 | | P15 | Yeah, there's lots of good ideas out there, there's just no money. |
| 6.4 | | P18 | Possibly because not many other people know that it's a problem so it hasn't been raised to them [Commissioners]. |
| 6.5 | | P16 | We need to demonstrate the impact and the value to individuals and the patient benefits and the system benefits as well because I'm sure they're huge. It's beyond my expertise to be able to go and explore that, but I do realise that that's one of the gaps. That's one of the areas that we'd need to do some work in. |
| 6.6 | | P30 | I think as we become less reactive and more proactive the smaller elements of the ophthalmology pathway like low vision we'll probably have more time to look at in more depth. |
| 6.7 | | P27 | Optometry is one thing but you've got dentistry, pharmacy and GPs, and you don't need me to tell you but historically optometry has not been looked at in the same way. So I think that that's probably why. |
| **Environmental Context and Resources: Practice resources** | | | |
| 7.1 | | P23 | most high street practices they won't even stock a LV aid. Right. So if someone comes in then what am I going to do, like what do I recommend? I can’t sell them anything because we don't have anything. |
| 7.2 | | P18 | not necessarily having the equipment to test them properly because you can't test at 6 metres or even at 3 metres on a projector chart and to actually change it to calibrate it for that one person's not worth it. |
| 7.3 | | P11 | Space, really. That could be another thing. Like I said, we're just having this refurbishment done, but again… (shakes head). |
| 7.4 | | P29 | And I also don't think sometimes the optoms have time to sit with a low vision patient and spend the time possibly necessary to explain sort of the eccentric viewing or to go through all the different magnifiers that are there and things like that. |
| 7.5 | | P02 | Plus the fact that unfortunately our practice, because it's in a very old building, has got loads of little steps in it, that would be a practical but very real barrier to a large low vision population coming in. |
| **Intentions: Passion** | | | |
| 8.1 | | P26 | They've just been doing it as a labour of love, and actually that is the phrase that one of them used, I do it because I love it. |
| 8.2 | | P15 | I walked in here and I was passionate about low vision. I joined my LOC [Local Optical Committee]. I joined a platform where I could do something about it. That was step one. So find someone passionate. |
| 8.3 | | P03 | When I was growing up there were several relatives who had serious disabilities, but that was part of our normality. … I think that also informs my wish to provide help and solutions and advice to people who are in more difficult circumstances. |
| 8.4 | | P16 | When you speak with people that are passionate about it, then individuals are absolutely very, very passionate. So I know that there's an appetite across the workforce, but I think it's sitting with a smaller number of individuals. |
| 8.5 | | P31 | I think what it seems to be is either you're fully passionate and you want to make a difference, or you've ticked the box at uni and that's it and now I'm never doing it again in my life, and there's not a lot in between. |
| 8.6 | | P22 | Part of it is because people just aren't that interested in what they do on a day-to-day basis. I think there's a little bit too much apathy. … So I think there's a general lack of interest in things like that. |
| **Beliefs about Consequences: Low vision outcome expectations** | | | |
| 9.1 | | P15 | I walk out of someone's house and they can read and before I walked in they couldn't read. They can see the TV, they can see to dial somebody, like it's life changing. It's literally life changing… I've just gone in and given them a device and it's changed their life. |
| 9.2 | | P22 | But a lot of the time with low vision it's just little life hacks that can make a massive difference. |
| 9.3 | | P06 | If you can just keep them a bit more independent and free and able to do their correspondence and cook for themselves and all that sort of thing, and they don't have to go into a home because they can’t cook, and if they can see just a bit more clear and have advice about lighting and contrast so they’re less likely to fall on their step |
| 9.4 | | P20 | I feel like when I think about low vision, I feel like I'm trying to make so much more of a difference than just sort of all these routine sight tests that you do. |
| 9.5 | | P09 | LVA work I think is quite soul destroying in that they're never going to be happy because what they want is their sight back so whatever you do for them isn't going to actually be good enough. |
| 9.6 | | P10 | I think it goes back to the confidence in the product again. So because we can't get them to see 20/20 anymore it's like, well am I willing to give it my all in selling you this electronic device that's going to help you when it might not? |
| 9.7 | | P24 | these people who do have low vision are very set in their own ways because they’re more of the older generation most people that we see so they don't like getting advice from a younger person. That's what I find. And also they don't like taking no for an answer, it's one of them. |
| **Beliefs about Capabilities: Confidence** | | | |
| 10.1 | | P28 | So I think having the confidence to discuss low vision and to discuss aids for people that have got low vision is a biggie really. I think if you've not got that confidence then you're not going to have that conversation. |
| 10.2 | | P15 | And some of the impressions are binocular vision is really difficult, low vision is really difficult. Like nobody knows why, someone started it and everyone says it |
| 10.3 | | P01 | But then I think low vision is one of those that you can make it as easy or as difficult as you want to for yourself. |
| 10.4 | | P24 | I think I've been scarred. I’ve got PTSD. … I think it's because I failed it so many times. I really do. I think it's because I failed it so many times. I really struggled with it. I just thought it was so hard. |
| 10.5 | | P02 | But I do remember it feeling quite overwhelming and intimidating because obviously the whole testing someone's vision in those situations is completely different and the conversations that you're having with patients are completely different. |
| 10.6 | | P23 | Like now I feel like I'm not fazed by any patient because I've dealt with them all and if it’s low vision I’m like OK I'm going to do this and I've got like a memory bank where I know, OK, this is what I need to do. |
| 10.7 | | P31 | I wonder if it's just that you're venturing into a path of unknown really …. I wonder if it's because you don't feel like you see low vision patients quite so often |
| 10.8 | | P10 | So that's why I did it and it did help the confidence. But then because we don't do it on a regular basis, I still don't feel confident like leading a low vision clinic because yeah, you learn about like the theory of which magnifiers to use, but it's so individual depending on the task at hand and what the patient has and what they need to be able to do |
| **Goals: Profitability** | | | |
| 11.1 | | P11 | I suppose the financial is, from a high street and business perspective, it's whether or not it's worth it for the business. That's how the high street will look at it. |
| 11.2 | | P22 | Is it a priority in practice? Probably not because it doesn't generate the revenue that the practices want to generate through other avenues really. And so, yeah, so I think that's the sticking point for me. |
| 11.3 | | P05 | No, it's just the money. It just comes down to the money at the end of the day. |
| 11.4 | | P27 | I think from a director point of view, the reason why we do it and continue to do it is because although you don't make any money from low vision as a business and that's the biggest hurdle, it's an incredibly good practice builder and that's our motivation behind it, as well as wanting to help the most visually deprived. |
| 11.5 | | P25 | the way we consider the sort of core issues would be like paediatrics and low vision, people who don't have that choice of how much money they want to spend on it and it's something that's become more of a basic requirement. So yeah, you don't ever want to profiteer off those people. |
| **Professional Role and Identity: Scope of practice** | | | |
| 12.1 | | P01 | Yeah, I mean it’s our bread and butter. If we strip the name of low vision out of it and we’ve got a patient coming in unable to do a function that they want to be able to do and you can give them an appliance that’s going to help them, well yeah, that’s what we’re looking to do every single day. |
| 12.2 | | P05 | But yeah, definitely I think it’s a primary care service, definitely. |
| 12.3 | | P04 | And then even now most of the opticians that I come across, they just see low vision as oh, that's something you signpost people to. |
| 12.4 | | P03 | The low vision care is actually almost a second level secondary service in that it's already been established that they have an eye condition, an eye disease, a progressive deterioration, so it's appropriate that that's dealt with at a higher level, at an ophthalmological level |
| 12.5 | | P17 | So as I have said all along that I think primary eye care services are best placed in my opinion to manage most of the run of the mill cases |
| 12.6 | | P21 | I think sort of basic prescription of simple magnifiers such as hand and stand magnifiers, doing a good refraction, prescribing higher additions if necessary, giving advice on lighting, signposting to charities, some advice on technology, I'm sure all that could really be done within high street optometry practice. |
| 12.7 | | P03 | But the over spectacles has worked and I think the fact that they can also be used just like sunglasses, the fact that they're a spectacle lens, I think fits more into an optometry practice than the magnifier. |
| 12.8 | | P16 | So parts of that service, that comprehensive service, parts of it might be delivered in the hospital eye service because that's where you've got your specialists. |
| **Reinforcement: Rewards** | | | |
| 13.1 | | P27 | When you see them and when you see them struggle and then you give them something to help, it sounds a bit cliche but it's literally amazing. |
| 13.2 | | P03 | But the people that we do help find it very, very useful and it's a personal satisfaction thing. |
| 13.3 | | P15 | I did low vision because for me as an optician it's the only time I feel I've made a difference to someone. |
| 13.4 | | P22 | I took a lot more interest in it in practice because you're using your skill set a bit more and you're actually providing a better quality of life for someone and you're finding solutions and solving problems that they come across day-to-day. So it's more rewarding in practice I think than just learning about it. |
| 13.5 | | P07 | And then it's lovely though, they’ll hand us in a box of chocolates or a bottle of whiskey or something that we register as a gift. It's really nice that they do that. That's not why we do it, but it makes you feel good if somebody thinks well, that was good, you really helped my mum or whatever. |
| **Emotion: Enjoyment** | | | |
| 14.1 | P07 | | But it was great fun. Have I made any money in low vision? Probably not. Has it been great fun along the way? Absolutely. |
| 14.2 | P03 | | What I enjoy at my work is the puzzle solving; each patient comes in, they've got a puzzle, they've got a particular challenge. |
| 14.3 | P20 | | It's sort of a bit more personal, like chatty sort of, finding out what they want help with… it's not quite as structured and it's much more personal in that sense, so that's probably a bit more enjoyable, just trying to figure out where you can benefit them. |
| 14.4 | P25 | | Yeah, I enjoyed it. I'm going to get all emotional. I fell into this profession as work experience. I had no idea what I wanted to do and 25 plus years later I'm still singing its praises and still learning new things and still just pushing because I like helping people. |
| 14.5 | P21 | | And people don't see it as being sexy enough in a way. If you've got somebody and you've diagnosed that they've got wet AMD and then you're doing injections that sounds so much better, or your monitoring glaucoma or writing out prescriptions, people like that. |
| 14.6 | P24 | | If I was being honest and you asked me why do you not think low vision is good, I would say because it's boring. That sounds awful to say that because it isn't boring when you delve into it, but to me it would just be a very boring and very difficult complicated subject. |
| **Emotion: Clinician wellbeing** | | | |
| 15.1 | | P05 | So I kind of feel like, oh, have I failed that patient? Have I not been persuasive enough? Yeah, I think that's one negative side of things. |
| 15.2 | | P31 | The feeling that you could get it wrong. But I think that's my personal thing is like you just don't want to fail |
| 15.3 | | P15 | What I found really difficult when I first started low vision and I almost gave it up, was I couldn't deal with like the emotional side of it. And some of the patients that came in and told me how they were living or what had happened to them, all their other circumstances besides sight loss. It really got to me in the first year or two. |
| 15.4 | | P02 | I’d say these days, having a little bit more experience, it really doesn't affect me emotionally too much. |
| 15.5 | | P25 | But yeah, there were some days that were harder than others. So I chose not to run like a whole day of clinic, so people would book to see me ad hoc like they would a dispensing clinic so I wasn't only doing that, so there wasn't like a heavyweight. |
| 15.6 | | P28 | Yeah, I think I'd have a discussion with my peers and speak to them about it, get their thoughts and feelings on it, and just talk with people about it to see if that will help shift it a little bit. |
